# An Evolutionary Cascade Model for Sauropod Dinosaur Gigantism - Overview, Update and Tests

**DOI:** 10.1371/journal.pone.0078573

**Published:** 2013-10-30

**Authors:** P. Martin Sander

**Affiliations:** Steinmann Institute of Geology, Mineralogy and Paleontology, University of Bonn, Bonn, Germany; Raymond M. Alf Museum of Paleontology, United States of America

## Abstract

Sauropod dinosaurs are a group of herbivorous dinosaurs which exceeded all other terrestrial vertebrates in mean and maximal body size. Sauropod dinosaurs were also the most successful and long-lived herbivorous tetrapod clade, but no abiological factors such as global environmental parameters conducive to their gigantism can be identified. These facts justify major efforts by evolutionary biologists and paleontologists to understand sauropods as living animals and to explain their evolutionary success and uniquely gigantic body size. Contributions to this research program have come from many fields and can be synthesized into a biological evolutionary cascade model of sauropod dinosaur gigantism (sauropod gigantism ECM). This review focuses on the sauropod gigantism ECM, providing an updated version based on the contributions to the PLoS ONE sauropod gigantism collection and on other very recent published evidence. The model consist of five separate evolutionary cascades (“Reproduction”, “Feeding”, “Head and neck”, “Avian-style lung”, and “Metabolism”). Each cascade starts with observed or inferred basal traits that either may be plesiomorphic or derived at the level of Sauropoda. Each trait confers hypothetical selective advantages which permit the evolution of the next trait. Feedback loops in the ECM consist of selective advantages originating from traits higher in the cascades but affecting lower traits. All cascades end in the trait “Very high body mass”. Each cascade is linked to at least one other cascade. Important plesiomorphic traits of sauropod dinosaurs that entered the model were ovipary as well as no mastication of food. Important evolutionary innovations (derived traits) were an avian-style respiratory system and an elevated basal metabolic rate. Comparison with other tetrapod lineages identifies factors limiting body size.

## Introduction

Dinosaurs of the clade Sauropoda were the largest terrestrial animals that ever lived [1], [2], [3]. They also were the herbivorous vertebrates that were predominant in terrestrial ecosystems for the longest time of any major clade, around 120 million years, from the Middle Jurassic to the end of the Cretaceous [4], [5]. Obviously, understanding their evolution and biology is a research program appropriate in size and importance to these extinct animals. The new millennium has witnessed an enormous growth in studies on sauropods, reflected by three edited volumes [3], [6], [7]. Since the interrelationships of major sauropod clades have largely been clarified (e.g., [8]), the focus has shifted to understanding sauropods as living animals and, through this, their remarkable evolutionary success and they evolution of their unique body size [1], [2], [3].

Scientists from many fields of biology and other backgrounds, sometimes far removed from traditional paleontology, have become interested in sauropods, recognizing them as models for understanding vertebrate evolution. Research has become increasingly quantitative and model-oriented. Starting with the simple quantification of sauropod body size in comparison with other clades of vertebrates [9], [10], [11], amazing progress has been made in quantifying dinosaur ecology [9], [11], [12], [13], [14], [15]. Modeling is worthwhile in sauropod research because, for one, sauropods went extinct 65 million years ago, making direct observation not an option, and also because of the great progress in computer applications and in the quantification and comparison of the biology of living animals and their ecosystems. The sauropod gigantism collection is meant to bring together current research on sauropods going beyond new finds in the field, beyond new phylogenies, and beyond new quantitative analyses of their fossil record. These areas of research, however, will remain as the foundation of research into sauropod gigantism.

### An evolutionary cascade model for sauropod dinosaur gigantism

Recently a new evolutionary perspective has been brought to understanding the uniquely gigantic body size of sauropod dinosaurs [2], an evolutionary cascade model (ECM) of sauropod dinosaur gigantism. This ECM posits that the evolution of sauropod gigantism was the result of the unique historical interplay of plesiomorphic (primitive) and derived traits, covering many aspects of sauropod biology, and selection pressure for ever larger body size [2]. There are two important premises to the sauropod gigantism ECM: for one, that sauropod gigantism as an evolutionary phenomenon was made possible by intrinsic, biological factors alone, without the need to hypothesize an influence of extrinsic abiotic factors and, second, that there is selection for large body size in terrestrial tetrapods.

The ECM was the focus of the second International Workshop on Sauropod Gigantism at the University of Bonn, Germany, in December, 2011. The workshop brought together a broad expertise on the subject, much of which is reflected in the current collection. In addition, research on sauropod dinosaurs and their gigantism continues at an amazing rate of discovery and of new insights, continuously expanding and testing the ECM. Such research includes both conventional paleontological work but also much innovative transdisciplinary work, showcased at the workshop as well as in this collection.

**Figure 1:**
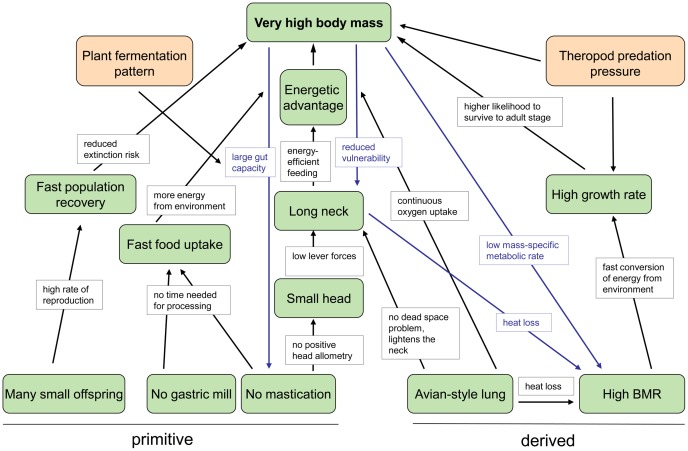
Original evolutionary cascade model (ECM) of sauropod gigantism. The model consists of five cascades that all end in the trait “very high body mass”. The green boxes contain the traits of sauropods, and the black arrows indicate selective advantages. Theropod predation pressure is depicted as a representative selection factor for body size increase. The ECM also incorporates evolutionary feedback loops (blue arrows). The blue boxes indicate the selective advantage in the feedback loop. BMR, basal metabolic rate. From [2].

The ECM is subdivided into of a series of evolutionary cascades [16], [17], each starting with a fundamental biological trait and ending in large body size (Fig. 1). Traits may either be observed or will have to be inferred, particularly in the case of fossil organisms. Each hypothesized trait, selective advantage, and feedback loop in the ECM is testable by new research, ranging from the discovery of new fossils and the development of sophisticated biomechanical and ecological models to phylogenetic tests of trait correlation.

The major purpose of this review paper is to test the sauropod gigantism ECM based on pertinent research published since late 2009 and in the current collection, and to present a refined version of the ECM. The review paper is also intended as an update of the Sander et al. review [2] that was published online on March 13, 2010. The 2010 paper [2] also reviews the pre-2009 literature, only the most pertinent of which is cited here again. Note that it is not the aim of this review to explore the history of paleobiological hypotheses about sauropods.

Many points that were expressed as hypotheses in the 2010 review paper [2] have now been tested and could not be falsified. In fact, the last three years saw a flurry of new studies, some of which were combined into a single volume [3] and have led to the general awareness that understanding sauropod gigantism is also of great value in understanding the limits of body size in terrestrial vertebrates in general.

This review paper's final function is to serve as an introduction to the Sauropod Gigantism Collection of PLOS ONE.

### Evolutionary cascades and ECMs

Evolutionary cascades are hypotheses of sequentiality and cause and effect. An evolutionary cascade consists of a sequence of biological traits in which one trait is hypothesized to have been the prerequiste for the evolution of the next one, driven by selection. As stated by Westneat [16] “Opportunity for selection caused by one trait leads to evolution of a response trait, which in turn creates a new opportunity for selection, driving the evolution of a new response trait”. These traits can be either plesiomorphic at the level of the clade in question or represent evolutionary innovations, forming a synapomorphy of the clade. Although the application of the evolutionary cascade concept has been remarkably widespread across groups of organisms, from bacteria [17] to sexual selection in birds [16], it is not yet widely used in organismal evolutionary biology.

The concept of evolutionary cascade is related to that of evolutionary constraint [18], [19] in two ways. An evolutionary cascade may result from the effects of several constraints arranged in a specific sequence, but an evolutionary cascade may also result from breaking one or more constraints by key innovations. The concept of evolutionary cascade thus seeks to go beyond the simpler concept of evolutionary constraint. Similarly, the concept of evolutionary cascade reaches beyond the concept of key innovation because it identifies multiple primitive traits, key innovations, and causations that shaped the evolutionary history of a group. All of these concepts have a historical perspective in common, explaining a pattern that is observed, usually over geological time scales. This perspective should not be confused with the experimental and process perspective commonly employed in the evolutionary biology of extant organisms.

Several cascades and their interplay have affected the evolutionary history of a clade. These cascades and their interplay may be described and visualized in an evolutionary cascade model such as the one for sauropod gigantism. An evolutionary cascade model is a tool that reveals the complex interplay of evolutionary constraints and historical contingencies that have allowed a lifestyle or trait to evolve. An ECM is thus a framework that explains the success and peculiarities of an animal lineage, independent of whether it is fossil or living. The nature of evolutionary cascade models, like that of all models, is heuristic, bringing interactions and constraints in an evolving lineage into sharper focus. In addition to traits and selection pressures acting on them, evolutionary cascade models can include feedback loops, making such links self-amplifying (Fig. 1). Note that an ECM essentially is a flow diagram, not a network diagram. This is unlike the correlated progression concept of Kemp [20], in which links between different traits are hypothesized but neither sequentiality nor causation of traits are addressed.

### Testing ECMs

**Figure 2:**
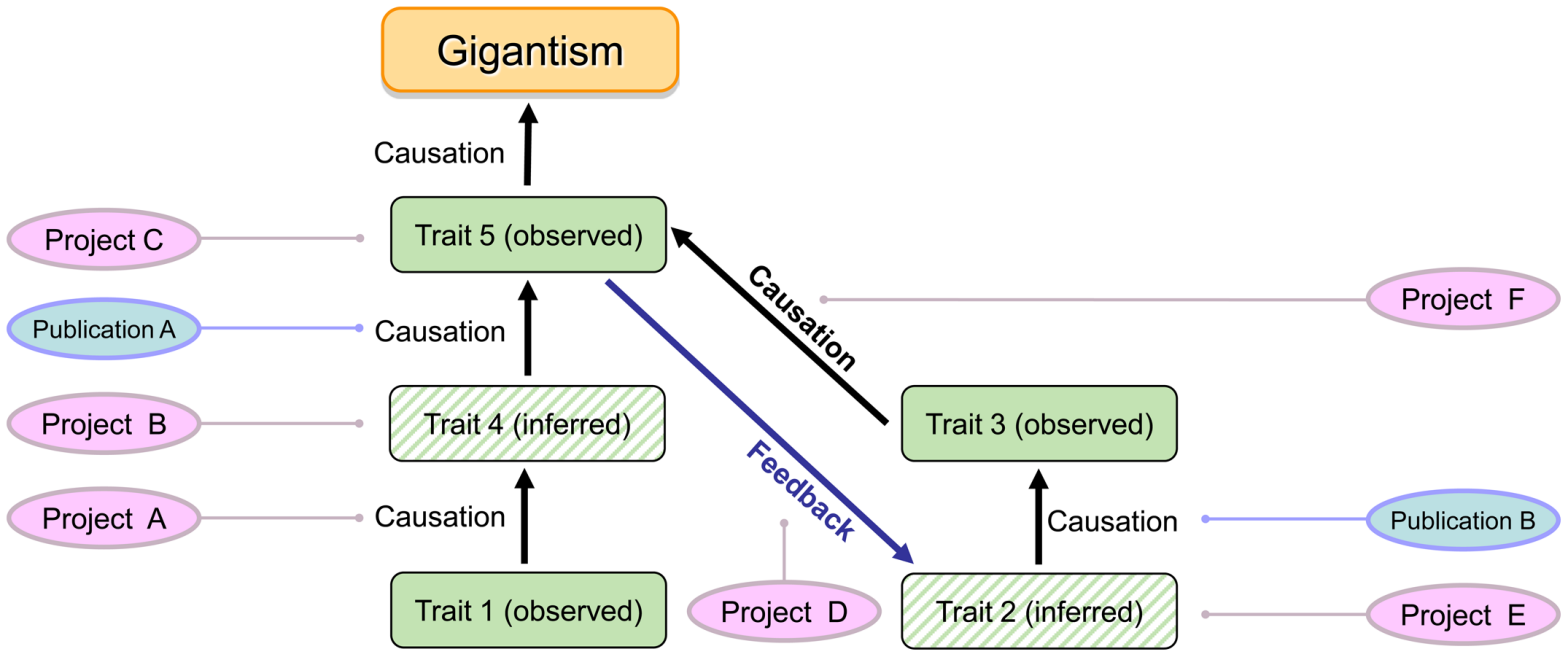
Testing an ECM by testing inferred traits and hypotheses of causation through transdisciplinary paleobiological research. Note that tests may consist of research projects designed for the specific purpose of falsification, come from published studies, and also may employ phylogenetic approaches.

Testing an evolutionary cascade model consists of testing its components, i.e., observed and inferred traits, evolutionary causations (i.e., selective advantages), and feedback loops. Inferred traits can be falsified by research specifically directed at this trait or by published evidence (Fig. 2). The same approach applies to hypothesized causal relationships, i.e., selective advantages and feedback loops. If the majority or all of the traits, selective advantages, and feedback loops are unfalsified, the ECM has passed the initial test and greater confidence can be placed in it. However, the predictions of the ECM must continually be tested, and the model modified, and ideally simplified, accordingly.

## Update on Sauropod Evolution and Paleobiology

### New taxa, finds, and phylogenies since 2009

#### New taxa

New sauropod taxa continue to be found or recognized through taxonomic work at a fast rate, underscoring the importance of sauropods in terrestrial ecosystems of the Jurassic and Cretaceous. While Mannion et al. [4] gave an early 2010 census of 175 valid genera, this number is up to 204 in early 2013, according to the Paleobiology Database (www.paleodb.org). There are no specific trends regarding where this new material comes from, but South America probably is the leader in diversity increase, the majority of new taxa pertaining to titanosaurs. Disparity does not seem to have increased markedly through these discoveries. Here I do not offer a comprehensive review but highlight only a few important finds, particularly those extending geographic and temporal ranges.

*Tapuiasaurus macedoi* from the Early Creatceous (Aptian) of Brazil [21] preserves the oldest typical titanosaur skull, indicating that advanced titanosaurs had evolved 30 million years earlier than previously believed. *Atacamatitan chilensis* from the Late Cretaceous of the Atacama Desert, Chile, is the first named sauropod from the western side of the Andes [22]. Likewise, the basal somphospondylian *Angolatitan adamastor* is the first sauropod from Angola and one of the few known from the Late Cretaceous of Africa [23]. Its Turonian age combined with its basal position in the cladogram suggest that *Angolatitan* may have been a relic form [23].

Already diverse sauropod faunas have become even more diverse, with a new diplodocine from the Late Jurassic Morrison Formation of northern Wyoming described as *Katedocus siberi* [24] and new titanosaurs from the Later Cretaceous of Patagonia, Argentina, such as *Elatitan lilloi* [25] and *Narambuenatitan palomoi* [26]. Bone histology indicates that the Morrison Formation species *Suuwassea emilieae* is a valid taxon because is not a juvenile of another Morrison Formation taxon [27] and phylogenetic analysis indicates it to be a dicraeosaurid [27], the first from North America. Particularly, the Morrison Formation taxa raise the question again about true sauropod diversity in this, the most species-rich of all sauropod-bearing formations.

#### New finds

Not only new taxa, but new discoveries and reanalyses of known taxa may be relevant for our understanding of sauropod biology and gigantism. A case in point is the putative early theropod dinosaur *Eoraptor* from the Carnian (Late Triassic) Ischigualasto Formation of Argentina This small biped turns out to be one of the most basal sauropodomorph dinosaurs instead, consistent with the sistergroup relationship of theropods and sauropodomorphs [28]. No later than the early Late Jurassic, sauropods had reached gigantic proportions as indicated by the remains of a mamenchisaurid from the Shishugou Formation of western China that include an ulna that is over 1 m long [29], indicating a humerus of around 1.5 m [27] and suggesting a femur of around 2.2 m in length. The large long bone shafts from the classical Late Triassic English locality of Aust Cliff remain enigmatic and cannot be assigned to Sauropoda [30]. At the other end of the stratigraphic column and the cladogram are the remains of gigantic individuals of the Maastrichtian titanosaur *Alamosaurus* from New Mexico [31], [32], comparable in size to the Argentinian giant titanosaurs *Argentinosaurus*, *Futalongkosaurus*, and *Puertasaurus*. These new finds [29], [31], [32] underscore the early evolution of giant sauropods no later than the Middle Jurassic and their later ubiquity, already apparent from the giant sauropods *Turiasaurus* (Late Jurassic, Spain), *Paralitan* (Early Cretaceous, Egypt), and *Sauroposeidon* (Early Cretaceous, USA), in addition to the giant Argentinian taxa mentioned above (see review in [2]). At the other end of the size spectrum, the island dwarf *Europasaurus* from the Late Jurassic of Germany continues to surprise in that the material from the type locality, and only geological horizon represents growth series of two morphs [33]. The morphs differ in final size, and previous body mass estimates of 800 kg apply to the large one [33]. Note that body mass estimate of “<5 t” by given Wilson & Curry Rogers [34] is misleading. It is uncertain whether the two morphs of *Europasaurus* represent different populations or species separated in time or possibly sexual morphs. Sauropod dinosaurs are now known from all continents, with a first record from Antarctica, a titanosaur tail vertebra having been described in 2012 [35].

#### New phylogenies and the emergence of the sauropod body plan

**Figure 3:**
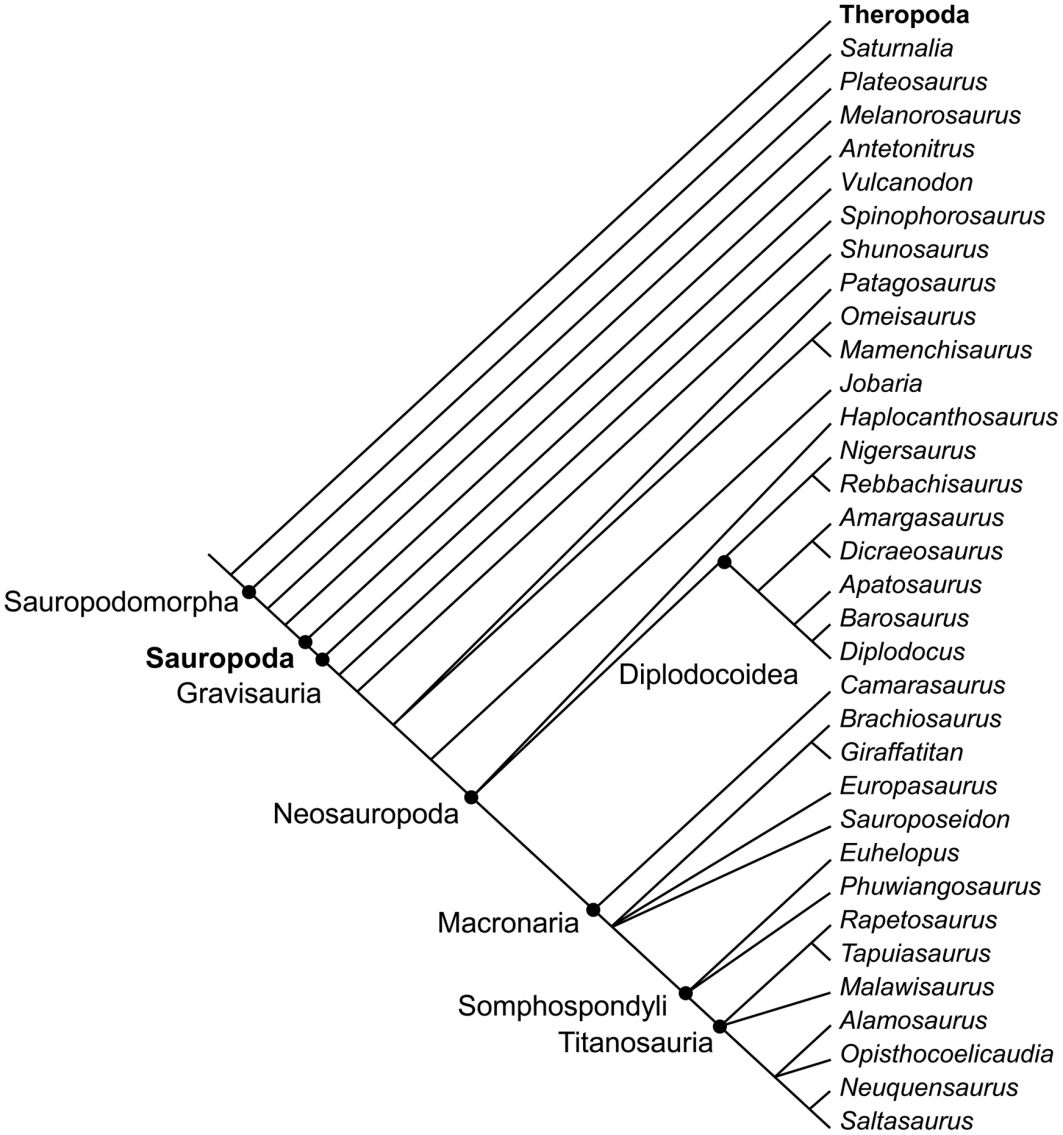
Simplified consensus phylogeny of Sauropoda at the genus level, containing only the best known and complete genera. Based on information in [21], [33], [36], [38], [39], [40], [188]. Dots indicate higher taxa. Note that no distinction is made between node-based and stem-based taxa.

The part of the sauropodomorph tree (Fig. 3) crucial for understanding sauropod gigantism is in the transition from derived non-sauropod sauropodomorphs to Sauropoda. Among sauropodomorphs, Yates et al. [36] recognize an obligatorily quadrupedal clade consisting of Melanorosauridae and Sauropoda, with *Antetonitrus* being the most basal sauropod. Sauropoda are defined as “the most inclusive clade containing *Saltasaurus loricatus* but not *Melanorosaurus readi*” [37]. Closer to the traditional concept of Sauropoda, before the intermediate forms such as *Antetonitrus* were known, is the taxon Gravisauria, which is defined as “the least inclusive clade containing *Vulcanodon karibaensis* and *Saltasaurus loricatus*” [37]. In Gravisauria, the typical sauropod body plan and all characters and traits relevant to the discussion of sauropod gigantism had evolved. Body size appears to increase to typical sauropod size in Gravisauria, but the rate of this increase is difficult to quantify because of the fragmentary nature of large basal and/or early sauropods. This prevents us from optimizing body size on the sauropod phylogeny at a higher resolution than was done before [2], because only smaller taxa are represented in the phylogeny.

While the phylogenetic relationships of the major sauropod clades to each other have been pretty well understood for the last 15 years [8], the ingroup relationships of Macronaria and particularly titanosaurs *sensu lato* have been difficult to resolve (Fig. 3). This situation is improving with recent analyses [21], [33], [38], [39], [40]. While these analyses differ in important details, they generally recover a monophyletic Brachiosauridae, different clades of basal titanosauroids, and well constrained Titanosauria.

Also, with the description of new taxa, hypotheses of their relationships are needed, which in turn improves our understanding of specific branches of the sauropod tree as well as its overall topology. A case in point is the study by Carballido et al. [41] on *Comahuesaurus*, which also resolves the interrelationships of Rebbachisauridae. A very similar topology but with fewer taxa was found by Mannion et al. [40]. The relationships of Diplodocoidea were recently reanalysed by Whitlock [42], including the largest number of taxa considered so far.

### Evolution and extinction

Our current understanding remains that gravisaurian sauropods first appear in the Late Triassic (Norian) but only become the dominant terrestrial herbivores in the Middle Jurassic after the extinction of non-sauropod sauropodomorphs [4]. The major clades of neosauropods (Diplodocoidea and Macronaria) originated in the Middle Jurassic, and already outside of these clades, gigantic forms evolved among Turiasauridae and Mamenchisauridae [29], [43]. The Late Jurassic saw the greatest diversification of the Diplodocoidea while the Early Cretaceous record is dominated by basal macronarians. The discovery [21] of an advanced titanosaur from the late Early Cretaceous (125–112 mya) explains the previously puzzling global distribution of the group in the Late Cretaceous, and suggests vicariance as the explanation of this pattern. Titanosaurs seem to have undergone an opportunistic radiation in the middle of the Cretaceous instead of competitively replacing diplodocoids and basal macronarians, gradually substituting them as the landmasses drifted apart [21], [39]. This scenario is consistent with the lack of evidence for a mid-Cretaceous terrestrial tetrapod extinction event [5].

All sauropod dinosaurs went extinct at the end of the Cretaceous. An analysis of Late Cretaceous sauropod diversity in southwestern Europe indicates no decline towards the K/Pg boundary [44], which is in agreement with catastrophic extinction not driven by biotic interaction but by an extrinsic cause. Ecological modeling of dinosaur, including sauropod, size-specific competition based on the scaling and disparity between parent and offspring size now suggests a possible explanation of why the generally large, oviparous dinosaurs would have been more vulnerable to extrinsic causes of extinction [12] than the contemporary viviparous small mammals. The model shows that after an extrinsically caused population collapse, large dinosaurs failed to re-establish populations as opposed to mammals. Based on a case study from the Dinosaur Park Formation of Alberta, Canada [45], [46], the assumption of the model of a strong left skew of body mass [12] was questioned and explained as a bias in the fossil record against small dinosaurs instead. The global nature of such a bias appears unlikely because the Dinosaur Park Formation is not representative of other Late Cretaceous dinosaur-bearing formations. Before the K/Pg extinction event, only the northern part of North America lacked sauropods [47], the extreme size of which are central to the model. The bias hypothesis was also refuted by a new compilation of vertebrate body size distribution through time [9] that had not been published at the time of the discussion about the extinction modeling [12], [46], [47]. The modeling approach [12] thus lends credence to an extrinsic cause for dinosaur extinction such as the meteorite impact creating the Chicxulub structure in Mexico [48].

Seemingly, this hypothesis about dinosaur extinction [12] is contrary to the hypothesis of Janis & Carrano [14], [49] that ovipary made dinosaur populations less at risk of extinction than populations of mammals of the same body size. However, the two hypotheses do not necessarily contradict each other since one [12] is comparing coexisting mammals and dinosaurs, while the other [49] addresses the question of what limits body size in the two groups.

### Diversity and biogeography

The emerging picture of sauropod diversity and biogeography also continues to solidify with a number of recent studies directed at refining our view of the patterns. The following section, on ecosystems, will explore some of the causations of these patterns. The diversity of dinosaurs, including sauropods, is commonly expressed by the total number of genera, with a 2010 census noting 175 sauropod genera, 325 theropod genera, and 223 ornithischian genera [50]. While there have been estimates of the total number dinosaur genera that ever lived (3500[11], [51]), these may well be overestimates because of the limited comparability of mammalian and dinosaurian ecosystem structure: dinosaurian ecosystems were characterized by a great size disparity between neonate and parent, resulting in a lack of parental care and ontogenetic niche shifting. This was particularly true for sauropods [52], [53], [54], and one dinosaur species may have occupied several niches as the individuals grew through several orders of magnitude in body size [12], [52]. In a similar mammalian ecosystem, these niches would be occupied by different species, thus leading to a greater species diversity in the mammals compared to the dinosaurs [12].

Progress has been made in reconstructing sauropod diversity through time [4], with reliable estimates for most time bins (geological stages) but not all, for example, the Late Cretaceous. The discovery of *Tapuisaurus* serves as a reminder of the nature of the sauropod fossil record in that the major patterns of diversification are well understood but that the specifics of time and place are just now emerging. In the broader analysis of dinosaur diversity through time, a new study [5] suggests that dinosaur faunas on the northern continents were never dominated by ornithischian dinosaurs, contrary to long-held beliefs. The only exception that appears to be remaining is the Campanian–Maastrichtian faunas of North America. Thus, the statement that “many terrestrial ecosystems were dominated by sauropods” [2] probably has to be modified to “most terrestrial ecosystems”, underscoring the importance of understanding sauropod gigantism.

The limitations of extrapolating from present patterns to the Mesozoic may be shown by an analysis of latitudinal distribution of diversity in dinosaur faunas [55]. Unlike in the modern world, where the tropics are the centers of diversity, dinosaurs appear to have been most diverse at mid- to high latitudes in temperate climates. This signal is well expressed in sauropodomorphs, particularly in the southern hemisphere. This diversity pattern also correlates with land area and may be partially explained by the weaker climate gradient in the Mesozoic [55].

### Ecosystems

Improvements in our understanding of ecosystems inhabited by sauropod dinosaurs have come from two different sources: the direct evidence provided by paleontology (including paleobotany), geology, and geochemistry, and the comparison with modern, mammal-dominated ecosystems. Whereas the former is based on generalizing from case studies, i.e., specific sauropod-bearing rock formations, the latter takes the opposite approach, using general relationships in ecosystems that are consistent with the fossil and rock record.

Arguably the most important source of information about sauropod dinosaurs and their environment has been the Upper Jurassic Morrison Formation of the western United States [56]. Although often portrayed as a semiarid habitat with low “fern prairies”, this is difficult to imagine considering the energy needs of the sauropod population. Growing evidence for conifer-dominated forest vegetation in the Morrison Formation suggests a much more mesic habitat [57] that would have been able to support the sauropods so amply documented by their fossils. An alternative solution to the problem of “feeding your sauropod” in the semiarid Morrison basin is offered by cyclicity in Sr isotope geochemistry in sauropod teeth, suggesting annual migrations of sauropods to the highlands bordering the basin in the west, possibly to cope with seasonal food shortages [58].

These observations partially support (migration) and partially contradict (aridity) the assumptions made by the most refined effort to quantitatively describe a sauropod ecosystem [59], again that of the Morrison Formation. This study by Farlow et al. incorporates the greatest range of information on extant animals as well observations from deep time, thus incorporating both approaches; its goal being to estimate the population density of dinosaurian megaherbivores, primarily sauropods. Farlow et al. estimate that endothermic dinosaurian megaherbivores would have had densities of “a few tens” of individuals of all ages but only a few subadults and adults per square kilometer [59]. Counts for dinosaurs with an intermediate metabolism would have been up to an order of magnitude greater. Farlow et al. [59] make no explicit distinction between sexually reproductive animals and juveniles, but only distinguish between “large subadults and adults” and “others”. Making this distinction would be the first step in using the result of Farlow et al. [59] to estimate the density of sauropod breeding populations in models of population growth rates, e.g., [12], [14], [49].

Recent studies using the general ecological approach would suggest that limitations in food availability would have affected sauropod populations less than mammalian megaherbivore populations because of the much lower minimum population densities of the former [11], [12], [14], [49], [52], [59]. Low viable population densities could have been afforded by sauropods for two reasons: their ovipary [14], [49], [52] and the strong left skew of sauropod body mass distribution [9] combined with the scaling of basal metabolic rate (BMR) [11]. Estimates of density of sauropods in the environment [12], [52], [59] thus are an order of magnitude lower than observed in modern mammalian ecosystems. This low density, however, was combined with a herbivore biomass that, at least at the global level, may have been one or more orders of magnitude higher in dinosaur (mostly sauropod) ecosystems than in modern ecosystems [11]. This study, however, did not take the different ontogenetic stages of large-bodied species into account, although it discusses their effects [11].

From all of this work, it is becoming increasingly clear that the key to understanding dinosaur ecosystems is the great size disparity between neonates and adults, epitomized by sauropods (see also section Cascade “Reproduction”). Only when researchers fully embrace this difference between dinosaurs and mammals in their analyses, will a profound understanding of dinosaurian ecosystems emerge.

## Test of the Sauropod Gigantism ECM by New Evidence

### The evolutionary cascade model for sauropod gigantism

As originally proposed [2], the evolutionary cascade model for sauropod gigantism consists of three basal traits that are plesiomorphic at the level of Sauropoda and two basal traits that are derived (Fig. 1). The plesiomorphic traits are “Many small offspring”, “No gastric mill”, and “No mastication”. The derived traits are “Avian-style lung” and “High BMR”. These traits are at the base of five cascades, only one of which (cascade “Reproduction”) is completely independent of the others. The other four (“Feeding”, “Head and neck”, “Respiration”, “Metabolism”) are interconnected to varying degrees, with one basal trait “No mastication” feeding into two cascades (“Feeding” and “Head and neck”). The original ECM does not visualize the distinction between observed and inferred traits.

**Figure 4:**
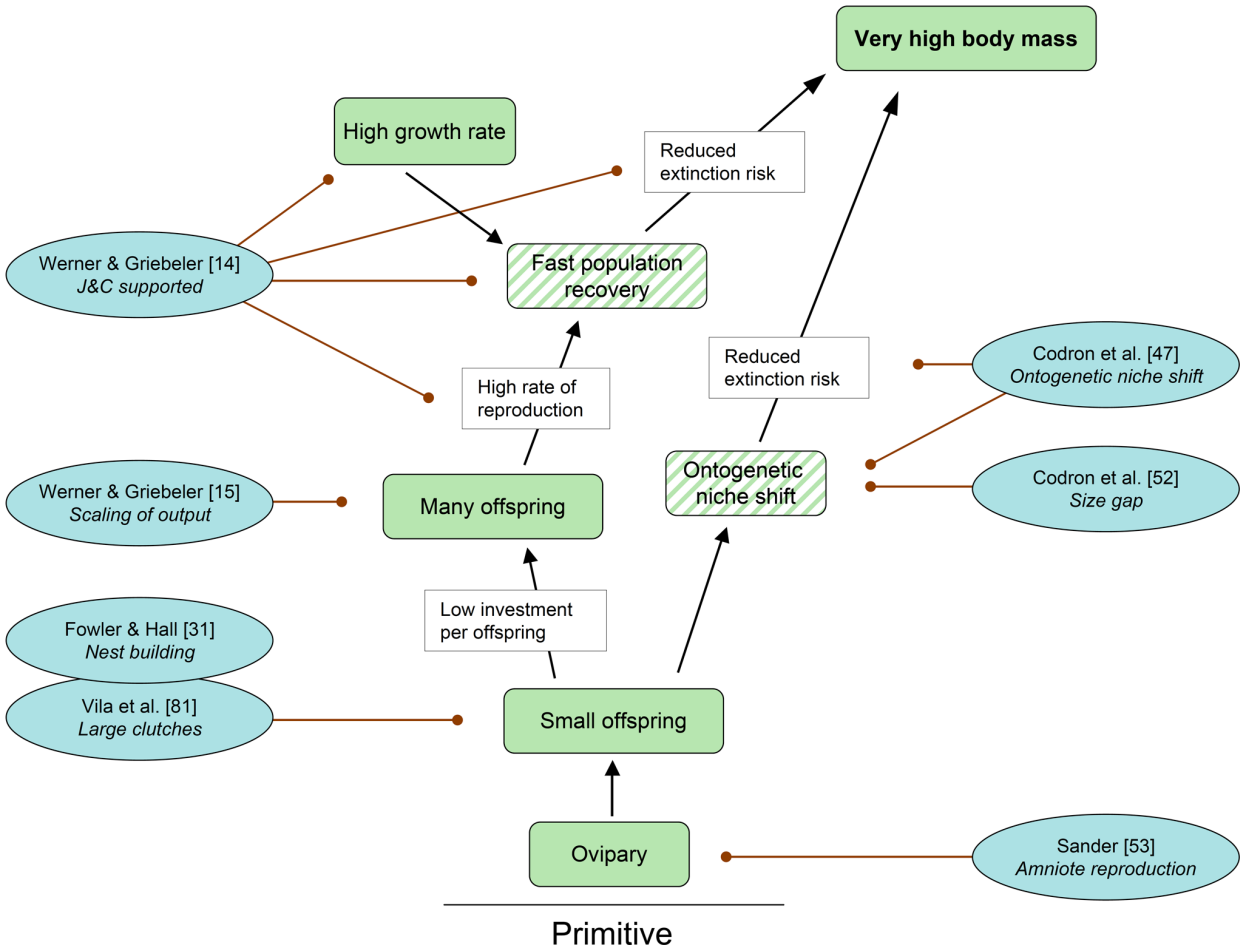
Cascade “Reproduction” with pertinent references published since 2010. Each reference includes a keyword indicating the aspect relevant to the cascade. Conventions used in this cascade are the same as in Fig. 1, except that a distinction is made between observed traits (solid color) and inferred traits (oblique stripes). The trait “High growth rate” is part of the cascade “Metabolism”. “J&C supported” stand for the Janis & Carrano hypothesis of dinosaur body size distribution [49]. See text for further explanations.

**Figure 5:**
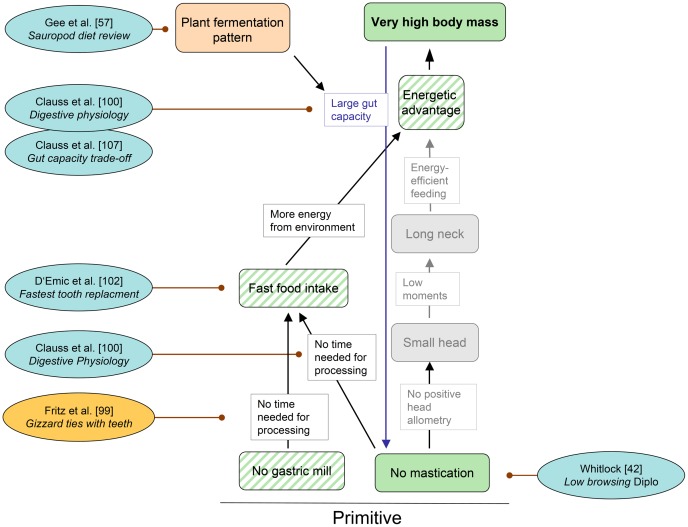
Cascade “Feeding” with pertinent references published since 2009. Each reference includes a keyword indicating the aspect relevant to the cascade. Conventions used in this cascade are the same as in Fig. 1, except that a distinction is made between observed traits and premises (solid color) and inferred traits or premises (oblique stripes). The orange references call the respective selective advantage into question. Grey indicates parts of another cascade that share traits with this one. See text for further explanations.

**Figure 6:**
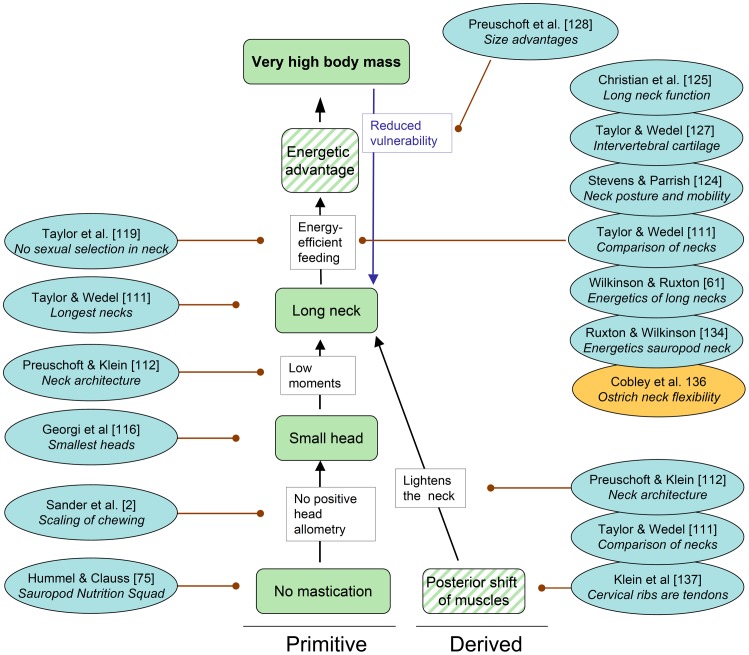
Cascade “Head and neck” with pertinent references published since 2011. Each reference includes a keyword indicating the aspect relevant to the cascade. Conventions used in this cascade are the same as in Fig. 1, except that a distinction is made between observed traits (solid color) and inferred traits (oblique stripes). See text for further explanations.

**Figure 7:**
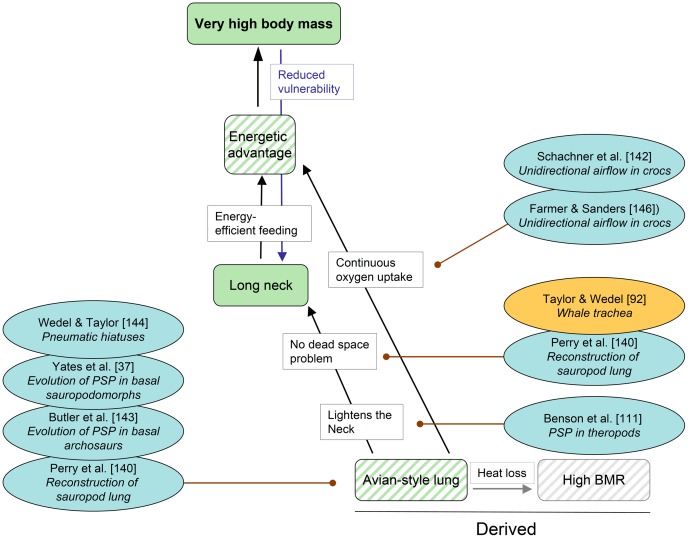
Cascade “Avian-style lung” with pertinent references published since 2011. Each reference includes a keyword indicating the aspect relevant to the cascade. Conventions used in this cascade are the same as in Fig. 1, except that a distinction is made between observed traits (solid color) and inferred traits (oblique stripes). See text for further explanations.

**Figure 8:**
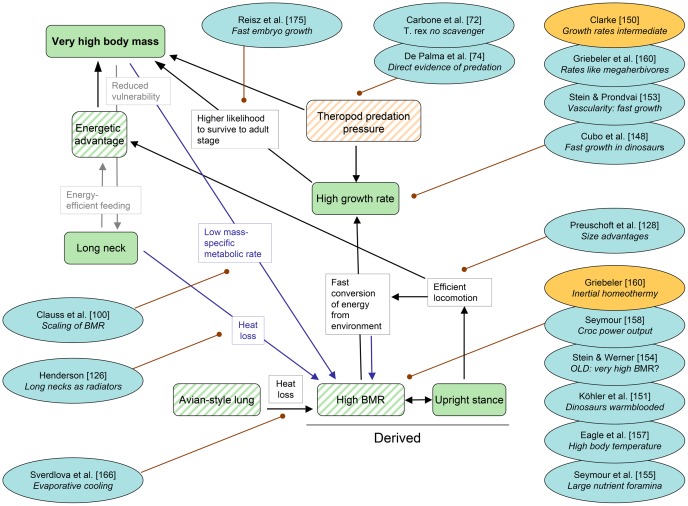
Cascade “Metabolism” with pertinent references published since 2011. Each reference includes a keyword indicating the aspect relevant to the cascade. Conventions used in this cascade are the same as in Fig. 1, except that a distinction is made between observed traits and premises (solid color) and inferred traits or premises (oblique stripes). Orange references call the respective trait into question. Grey indicates parts of another cascade that share traits with this one. Theropod predation pressure is an inferred premise. See text for further explanations.

The new evidence bearing on the sauropod gigantism ECM is organized topically within the individual cascade, going up each of the cascades from the basal trait to the final one, very high body mass (Figs. 4, 5, 6, 7, 8). Cascades consist of traits, hypothesized selective advantage,s and feedback loops. Unlike in the original model, an explicit distinction is made between observed and inferred traits. However, before each cascade is discussed, new developments regarding the premises underlying research on sauropod gigantism in general and the ECM in particular need to be addressed.

### Testing the premises

One of the basic assumptions of the ECM was that the evolution of sauropod gigantism is primarily under intrinsic control, meaning that it was driven by biological factors [2]. Extrinsic controls, such as changing global environmental parameters, were largely excluded from consideration in the ECM because those environmental parameters that are known or can be reasonably well inferred show no correlation with sauropod body size evolution [2]. This hypothesis of no correlation was tested by Sookias et al. [13] using maximum-likelihood analyses of Late Paleozoic to Jurassic terrestrial vertebrate evolution, and they showed that biological factors alone are sufficient to explain patterns of size evolution in dinosaurs [13]. The Cretaceous was not covered by this analysis [13], which should not be a problem in the current context because sauropod gigantism already had evolved in the Late Triassic and Jurassic. However, recently a specific hypothesis of extrinsic control by Midgley et al. [60], invoking raised levels of carbon dioxide during the Mesozoic to account for dinosaur gigantism, was resurrected [61] and awaits further scrutiny.

Among the several drivers of evolutionary body size increase in dinosaurs [9], [62], [63], also known as “Cope's Rule” [64], [65], [66], [67], predation pressure has received renewed attention. Ecological models suggest that in dinosaur ecosystems, there was a size threshold above which theropods could not subsist on prey much smaller than themselves but had to hunt prey of their own body mass [52]. This threshold, which is 21.5 kg body mass in modern terrestrial ecosystems [68], may have been 25 to 30 kg for dinosaur ecosystems [52]. This means that theropod predation pressure on sauropods must have been strong before the individuals exceeded the largest theropods in their habitat in body mass, as is the case in modern mammal ecosystems with the largest herbivores [69], [70]. At least in modern large-mammal ecosystems, the largest predators generally do not take prey that is significantly larger than themselves, not even by pack-hunting [68], [69], [70], [71].

Predation pressure by large theropods on sauropods also hinges on the question if such giants as *Tyrannosaurus* indeed were actively hunting their prey or if they only were scavengers. Models of carrion encounter vs. prey encounter support active hunting because large theropods would have been the last ones to have found any carrion which would have been consumed by smaller theropods and juveniles first [72]. Other lines of evidence that large theropods were active hunters were reviewed by Brusatte et al. [73]. The most recent addition to the discussion is direct evidence of predation [74]. However, abundance of *Tyrannosaurus* in the Late Cretaceous Hell Creek Formation of Montana (USA) suggests that at least adult tyrannosaurs may also have subsisted on carrion [75]. While scavening may have been a way of life in some large theropods, such as *Tyrannosaurus*, the sum of the evidence argues for large theropods generally having been active predators.

Predation pressure on herbivorous dinosaurs, i.e., ornithischians and sauropods, thus probably explains the strong left skew seen in body size histograms of these dinosaur groups [9], [12].

Traits of sauropod reproductive biology, i.e., the lack of parental care and the large number of small offspring, also must have resulted in increased predation pressure which in turn would have led to strong selection for larger body size. In particular, because unlike in modern meagherbivores no trophic energy was lost due to parental care [76], juveniles of even the largest herbivorous dinosaur species were available to predators. This provided the predators with a greater resource base compared to modern ecosystems, which would have facilitated larger predator body size [52], [76], raising the body size ante for sauropods even further. This effect was not limited to sauropods, of course, but would have influenced ornithischian-dominated ecosystems as well.

**Trait.** Very high body mass

This trait will be discussed first because all cascades culminate in it. The discussion of this trait only covers the most recent developments and literature because an in-depth review is found in Sander et al. [2].

Finds of exceptionally large sauropod individuals continue to be made (see above), making the trait “Very high body mass” immediately obvious. Compilations from the literature also drive home the point [3], [12]. Dinosaurs show little overlap with mammals in body mass to species richness plots and show a strongly left-skewed distribution compared to the strongly right-skewed distribution of extant and fossil mammals, with sauropods occupying the far right of the body mass spectrum [9], [12].

Much of the work underlying the ECM requires accurate estimates of body masses of sauropods at the level of the individual. Classically, two approaches have been taken for estimating body mass in extinct tetrapods: mass estimates based on body volume estimates and mass estimates based on scaling of long bone dimensions in extant tetrapods. The most general dataset compiled so far offers a universal scaling relationship of long bone circumference and body mass in tetrapods [10]. Values for sauropods calculated from this relationship are similar to estimates obtained by earlier workers, e.g., 35,780 kg for the Berlin skeleton of *Giraffatitan* [10]. Volume-based estimates also have became more refined such as the “minimum convex hull method” [77] which was calibrated using extant animals of known mass. This method resulted in a seemingly “low” estimate of 23,200 kg for the Berlin *Giraffatitan* [77].

A novel approach to “weighing” sauropods is using soil mechanics to estimate the mass of a trackmaker from the substrate deformation it caused [78]. Dinosaur tracks in a trackway always include a kinetic component in the forces that generated them in addition to the static component. However, in large slow-moving animals with columnar legs such as elephants and sauropods, the static component greatly exceeds the kinetic component. Thus, soil mechanical finite element models were calibrated for estimating sauropod masses by experiments with an elephant [78].

### Cascade “Reproduction” (Fig. 4)

**Trait (observed and inferred).** Many small offspring

Sauropod dinosaurs, like all extinct and living dinosaurs and all archosaurs, reproduced via ovipary, presumably being constrained to this mode of reproduction by their calcified eggshells [79]. This seemingly straightforward statement takes on a new meaning when one considers that the biomechanical upper limits to egg mass [52], [53], [71], [80], derived from work on bird eggs [71], [80], means that sauropod hatchlings must have been very small compared to the adult [12], [14], [52]. This is in accordance with the fossil record that shows that all known sauropod eggs had a volume not exceeding 5 liters [80], [81], [82] and that most were buried in the substrate [81], [83], [84].

The small size of the offspring relative to the adult led to the hypothesis [53] that large sauropods must have laid hundreds of eggs per year in several clutches to have a biologically realistic reproductive output. This hypothesis recently found support in a detailed analysis of scaling of egg mass, clutch mass, and annual clutch mass in the extant phylogenetic bracket of sauropods [15]. This study concluded that medium to large sauropods may have laid as many as 200 to 400 eggs per year, and smaller ones <200 eggs per year.

Particularly, the laying of several clutches and the size difference between hatchling and adult make any form of parental care unlikely. Lack of parental care is also suggested by the burial of the egg clutches by scratch-digging of the female sauropod [81], [83] as practiced by extant turtles [83]. Distribution of the annual reproductive effort, i.e., annual clutch mass [15], of large sauropods over several clutches per year is suggested by phylogenetic inference combined with scaling arguments [15] and by physiological arguments [85], both based on modern amniotes. Several clutches per year is consistent with the generally small clutch size [<15 eggs], a report of up 28 eggs per clutch [81] notwithstanding. This report [81] failed to test the hypothesis, using shell thickness, that such large egg clusters represent several superimposed or closely associated clutches. Different clutches of a single species of sauropod differ in shell thickness while eggs in a single clutch do not [53]. This kind of work on eggshell thickness variation should be extended to the extant phylogenetic bracket of dinosaurs.

The possible exception to the lack of parental care may be the unburied eggs from the Argentinian locality of Auca Mahuevo [53], [86], although other studied suggest burial of these eggs as well [87], [88] and thus lack of parental care.

**Selective advantage.** High rate of reproduction

Based on data for extant birds and mammals, an early, seminal study Janis & Carrano [54] had suggested that scaling of reproductive output with body mass differs fundamentally between extant birds and mammals, and that this is linked to the oviparous mode of reproduction in birds vs. the vivipary of mammals. The latter showed negative allomtery of number of offspring with body mass with increasing body mass [49], whereas birds show no decrease in reproductive output (but no increase either, i.e., no correlation) with body mass [49]. Recent analysis of a comprehensive dataset for extant birds and mammals by Werner & Griebeler [14] supports these observations, with birds showing a positive correlation between annual offspring number and body mass while mammals show a negative correlation. Werner & Griebeler [14] also noted that sauropod reproductive output was at the upper limit of that expected for a sauropod-sized bird and much higher than predicted for a sauropod-sized mammal, attributing this to the ovipary of sauropods.

**Trait (inferred).** Fast population recovery

Janis & Carrano [49] hypothesized that a high reproduction rate would allow fast recovery of a population after a population crash, and this benefit also would have applied to dinosaurs [49]. The inferred trait of fast population recovery recently found support in a simple mathematical model comparing population recovery rates in a large dinosaur and a large mammal, with the dinosaur population recovering much faster [14]. However, fast population recovery also depends on a high growth rate of the offspring [14], which is lacking in extant non-avian reptiles [89], [90]. Note that the trait “Fast population recovery” depends on a trait from a different cascade, the trait “High growth rate”.

**Selective advantage.** Reduced extinction risk

In the context of sauropod gigantism, a low reproductive output has been shown to increase the risk of extinction [14], as originally hypothesized by Janis & Carrano [49]. This will come as no surprise to a conservation biologist. Janis & Carrano [49] went on to hypothesize that reproductive output will introduce an upper limit to body size depending on reproductive output. Larger-bodied species will have lower population densities than smaller-bodied species, leading to a higher risk of population extinction through stochastic perturbations. Since the extinction risk decreases with increasing reproductive output, species with a higher reproductive output can have a larger body size than species with a lower reproductive output [14], [49]. This work should be extended by a comparative study of population recovery in real populations of mammals, birds, and non-avian reptiles, although likely there is much information on this subject already available in the conservation biology literature.

In sauropods, the selective advantage of a reduced extinction risk may also have resulted directly from the trait “Many small offspring”. The great size difference between hatchling and fully grown sauropods as a consequence of ovipary probably meant extensive ontogenetic niche shifting, with different life stages being adapted to different environmental conditions [12], [52]. This diversity of niches in a single biological species at different times in its ontogeny is hypothesized by Codron et al. [52] to mean that in times of environmental perturbations some life stages may have been less affected or even may have preferentially survived, making the species as a whole more resilient to such perturbations. This hypothesis should be tested by studies on extant reptiles with a great size difference between offspring and parent, such as large-bodied crocodile species and marine turtles.

### Cascade “Feeding” (Fig. 5)

**Trait.** No mastication

It is generally accepted that sauropod dinosaurs did not chew their food [91], [92], [93], and no evidence to the contrary has been published in recent decades. To a certain extent, lack of mastication may be a derived trait. Basal sauropodomorphs apparently possessed fleshy cheeks, a prerequisite for chewing, but fleshy cheeks were reduced in sauropods as an adaptation to bulk feeding [36]. The focus of investigations on the sauropod food gathering apparatus is now on the details of the functions of the dentition in different taxa, based on detailed descriptions of morphology and wear patterns of the dentition, macroscopic and microscopic tooth wear patterns, and muzzle shape [42], and finally biomechanical modeling using finite element analysis [92], [94]. Such work lends strong support to the notion that diplodocoid sauropods were low to mid-height browsers [42]. Both generalists and specialist were found among diplodocoid sauropods, with the low browsers possibly preferring a diet of horsetails [95]. However, our understanding of the functioning of the non-masticating feeding apparatus will remain incomplete without an explanation of the common finds of isolated tooth rows in many sauropod taxa, e.g., *Giraffatitan* [96]. Possibly, the tooth row was strengthened by a keratinous sheath that covered the exposed part of the roots as suggested for dinosaurs in general [97]. Such a sheath may or may not be homologous to the small lower bill that may have been present in some basal sauropodomorphs [28]. An improved understanding of the implications of the trait “No mastication” may come from experimental work on extant herbivorous reptiles. Herbivorous birds are not informative in this regard because they use a gastric mill to comminute plant matter instead of a dentition (see following section).

**Trait.** No gastric mill

In the absence of a chewing dentition, sauropod dinosaurs classically were believed to have processed their plant fodder in a gastric mill similar to granivorous birds [98]. The comparative analysis of ostrich feces and mammalian herbivore feces indicates that a gastric mill is as effective in particle size reduction as a chewing dentition [99]. However, multiple lines of evidence based on observations on extant birds make it unlikely that sauropods possessed a gastric mill [98], including the rarity of potential gastroliths found with seemingly complete sauropod skeletons compared to their consistent presence and significant mass in herbivorous birds (approx. 1% of body mass [98]).

**Selective advantage.** No time needed for processing

The selective advantage of not reducing fodder particle size is that no time is needed to do so. Time needed for chewing scales positively with body mass in extant mammals [69], [100], limiting mammalian herbivore body size to a mass of about 18 t, at which the animal would have to spend 24 hours a day feeding [69], [100]. Even if this scaling relationship for extant mammals may not have applied to chewing dinosaurs such as hadrosaurs and ceratopsians, it is likely that chewing would have limited their body size as well.

While similar data about scaling of duration of gastric mill use s are lacking for birds, we cannot be sure that particle size reduction in a gastric mill limits body size. However, all birds and non-avian dinosaurs that have a gastric mill are small (dinosaurs, >25 kg) or medium-sized (birds, >250 kg) [99], suggesting other limitations to their body size. Contrary to the suggestion by Sander & Clauss [1] and Sander et al. [2], the lack of a gastric mill thus may not have been a prerequisite for sauropod gigantism.

**Trait (inferred).** Fast food intake

Food intake rate can only be observed in extant animals, but a high food intake rate has been inferred for sauropod dinosaurs for two reasons [1], [91], [101]: lack of mastication and high energy demand. The hypothesis of fast food intake can be tested by quantifying tooth wear which should increase with intake rate. Indeed, the common Morrison Formation sauropod *Diplodocus* has recently been shown to have the second-highest tooth replacement rate known among archosaurs [102]. Based on the analysis of overlapping daily growth increments in successive replacement teeth, replacement rates on the order of 35 days are reconstructed for *Diplodocus* [102]. Approximately 62 days were estimated for *Camarasaurus* [102], which is bracketed by the rates for hadrosaurs. The highest rates (“less than 30 days” [103], now refined to “15–30” days [102]) had previously been reported for *Nigersaurus* but it was not known whether this was representative for sauropods in general because of the extremely modified dentition of this taxon [103]. The new study [102] suggests that all neosauropods at least had such high tooth replacement rates, indicating fast tooth wear. Because of the small size of sauropod teeth compared to the bulk of their bearer, such high replacement rates may not be entirely surprising but clearly indicate extreme abrasion of teeth. Unlike grasses and with the exception of horsetails, Mesozoic sauropod food plants were not particularly abrasive [95], suggesting high intake rates as the explanation. Although grass phytoliths were discovered in putative sauropod coprolites from the Late Cretaceous of India [104], the sauropod affinitiy of these coprolites cannot be established [105], [106]. A comparison of sauropod tooth abrasion rates with those of functionally analogous non-chewing teeth (i.e., incisors) of herbivorous mammals should be done to further test the hypothesis of fast food intake.

**Selective advantage.** More energy from the environment

Provided that plant resources are not limited in the environment, an animal with a greater capacity for food intake rate will be able to take up more energy from the environment that an animal with a lower capacity [100]. This is supported by empirical data on extant mammals, reviewed in [100]. This increased energy taken up from the environment translates directly into an energetic advantage.

**Trait (inferred).** Energetic advantage

Four evolutionary cascades end in this trait, indicating that at least four traits contributed to the energetic advantage permitting sauropod gigantism, but the trait per se has not received further comparative study in extant or extinct animals since the sauropod gigantism ECM was formulated.

**Feedback loop.** Large gut capacity

In the original version of the ECM, a feedback loop leads from the trait “Very high body mass” to the trait “No mastication” [1], [2]. This feedback loops, called “Large gut capacity” posited that very high body mass is favored by the positive scaling of the retention time of the ingested food in the gut, based on data from extant animals [107], [108]. This would have allowed sauropods to compensate for the lack of mechanical breakdown of their fodder by increasing food retention time [107], [108], leading to greater digestive efficiency in large-bodied dinosaurs, following the Jarman-Bell Principle in extant animals [69]. This idea was supported by the isometric scaling of gut volume compared to the negative allometry of energy requirement. However, recent work [100], [109], [110] called the hypothesis of positive scaling of ingesta retention time in extant animals into question because of the lack of empirical data, which instead tend to show that food retention time is independent of body mass. Accordingly, other factors than scaling of digestive physiology may have facilitated sauropod gigantism [100].

Nonetheless, isometric scaling of gut capacity would have generated the feedback loop “Large gut capacity” because of the negative allometry of BMR, but the feedback loop is probably weaker than originally envisaged. With an isometric increase in gut volume, larger animals can digest more food at the same time and thus subsist on lower-quality forage. Sauropods would have needed excessively large guts to compensate for the lack of particle reduction. In fact, the sauropod body cavity appears to have provided sufficient space for such large guts [100].

### Cascade “Head and neck” (Fig. 6)

**Trait.** No mastication

The observed trait of no mastication has been discussed above. In addition to the selective advantage of “No time needed for food processing”, this trait provides a crucial selective advantage associated with the sauropod neck [111], [112].

**Selective advantage**. No positive head allometry

Because of the scaling effects surrounding mastication, extant masticators show positive head allometry [2], and this may have applied to masticating dinosaurs as well, as suggested by the scaling of skull size in ceratopsian dinosaurs [113]. This is because chewing performance scales with the second power, while body mass scales with the third power. The reason for chewing performance scales with the second power is that chewing performance is determined by two surface areas: that of the combined tooth grinding surface and that of the chewing muscle cross section (the power of a muscle being determined by its cross section, not its volume), The positive head allometry of chewing herbivores resulting from these scaling effects is weakened by the negative allometry (exponent of 0.66 to 0.75) of energy demand, i.e., BMR, to body mass, well known from extant animals (for a discussion of this scaling relationship, see [100]). Nevertheless positive head allometry appears to the inescapable effect faced by any chewer, as seen, e.g., in the ontogeny of the hadrosaur *Prosaurolophus* [114]) and in horse evolution [115].

**Trait.** Small head

Sauropod dinosaurs had the relatively smallest heads in length and mass of any non-avian dinosaur [113], [116] and likely of any terrestrial tetrapod, although comparative data across extinct and extant Tetrapoda have not been compiled. The small head of sauropods had to serve three major functions, the space required for all of which apparently shows a negative allometry with body mass. These functions are: food intake, housing of the sense organs, and housing of the brain and inner ear. The relatively very small brain of sauropods [117], [118] stands in stark contrast with many other aspects of sauropod biology, such as their high BMR, and remains enigmatic.

**Selective advantage.** Low moments

The obvious selective advantages of a small head are the low moments of force that it bestows on the neck [112], permitting a longer neck than would be possible with a larger head [111], [112]. The importance of moments of force in the biomechanics of long-necked mammals and birds has received much attention and most recently has been reviewed by Taylor & Wedel ([111], but see also [119], [120], [121], [122], [123], [124]).

**Trait.** Long neck

The defining feature of sauropod dinosaurs, their uniquely long neck, received a thorough review by Taylor & Wedel ([111], see also [119], [120], [121], [122], [123], [124]). This review includes a list of traits making the evolution of the long neck possible, most of which are derived from comparison with extant animals [111]. This list contains the ones discussed in depth here, as well as some more general and obvious traits such as large body size, quadrupedal stance, a phylogenetically flexible number of cervical vertebrae (unlike in mammals that are constrained to seven cervicals), and elongation of the cervical vertebrae [111].

Considering the importance of the neck, this collection contains no fewer than four contributions on the subject [112], [125], [126], [127], including detailed studies on the osteology and posture of the neck [112], [125], [127]. Based on various lines of evidence, the majority of studies suggest a diversity of neck postures in sauropods, from steeply inclined to horizontal, depending on taxon. Articulation of fossil necks in the osteologically neutral pose, on the other hand, suggests a subhorizontal neck posture for all sauropods [123], [124]. The topics of neck posture and flexibility will be revisited below from the perspective of the major selective advantage provided by the long neck, i.e., the selective advantage “Energy-efficient feeding”.

Although neck length would have been constrained by mechanical factors [111], [112], [128], the question has recently been raised whether there were neuroanatomical constraints as well, i.e., the travel times of nerve signals from the tip of the tail to the brain [129]. Signal travel times must have been up to half a second in a large sauropod based on the comparison with extant animals. Since the connection between brain and tip of tail is established by a single nerve cell, cell size might have posed an upper limit to sauropod body size [129].

**Selective advantage.** Energy-efficient feeding

The central hypothesis of the ECM possibly is that the long neck of sauropods facilitated highly energy-efficient feeding, both by giving access to tall vegetation and by extending the reach of the head without moving the heavy body. While it is clear that a longer neck confers advantages to an animal of any size [2], [61], [128], as shown by studies on extant animals [61], the important point with regard to sauropods is that this advantage favorably scales with body mass. The scaling effect lies in the scaling of acceleration and deceleration of the body because larger animals are less “athletic” than smaller ones because muscle power only increases with the square of linear size whereas mass increases with the third power (see reviews in [130], [131]).

A premise of the hypothesis of energy-efficient feeding is that the main function of the long neck indeed was feeding and not some other function in physiology, reproduction or behavior. In particular, the hypothesis that sauropod neck elongation was a result of runaway sexual selection [132], as had been hypothesized for giraffes [119], can now be rejected [119].

Several kinds of new model calculations, on the other hand, do support the hypothesized selective advantage ([61], [128], [133], [134], see also [135]). Model calculations addressing high browsing based on *Euhelopus* and *Giraffatitan* [133] indicate that the energetic advantage of this design outweighs its metabolic costs (i.e. raising the neck and supplying it and the head with blood). Model calculations specifically addressing low browsing in sauropods [61], [128] also confirm the hypothesis that the long neck greatly reduced the need for the animal to change its location during feeding. This would have resulted in energy savings of 80% in a *Brachiosaurus* bearing a nine-meter neck compared to a minimally-necked one [61]. Both studies [61], [128] independently concluded that the energetic advantage of neck length levels off eventually with increasing neck length. The energetic advantage is particularly apparent if target vegetation has a patchy distribution as shown by a case study on the relatively longest-necked sauropod, *Mamenchisaurus* [125]. Therefore, there is strong support for the hypothesis that the long neck of sauropods provided a major energetic and thus selective advantage in feeding efficiency.

While both an erect and a horizontal neck convey major energetic advantages, the crucial question of neck flexibility is still surrounded by controversy [111], [123], [124], exemplified by papers in this collection [125], [127] and another recent one [136]. The flexibility of the neck, which particularly in the low-browsing posture determines whether the animal can browse on a volume or only a large surface area, with the obvious implications for feeding efficiency. Neck flexibility was constrained by the long cervical ribs in most sauropods except diplodocoids. Diplodocoid sauropods had evolutionarily reduced the long posterior process of the cervical ribs so that they do not extend across intervertebral joints, which would have increased neck flexibility [111], [112], [125], [137], [138].

Virtual articulation of neck vertebrae and simplified models suggests that sauropod necks were held largely horizontally and may not have been flexible enough to cover a volume but only a surface [123], [124]. Similarly, physical articulation of a *Mamenchisaurus* neck and optimization of intervertebral articular surface pressure indicate a horizontal posture and partitioning of flexibility along the vertebral column, with a relatively stiff middle neck region [123], [124], [125]. However, the same methodological approach concludes that basal marcronarians held their necks at a steep angle [123], [124], [125].

A full understanding of sauropod neck posture and flexibility is hampered by the need to reconstruct the thickness of the cartilage covering the intervertebral joints and the zygapophyses [127], [136]. With mammals and crocodiles generally having thicker cartilage than birds, the choice of either of these extant taxa for comparison results in either a more flexible or less flexible neck. Evidence from successive sauropod neck vertebrae fossilized in articulation suggests relatively thick cartilage covers and thus flexible necks [127]. The discrepancy in the results of these studies [112], [123], [124], [125], [127] make sensitivity analyses of neck posture advisable, quantifying the effect of different hypothetical cartilage covers on flexibility and resulting feeding volume. Also, more necks preserved *in situ* should be studied to address the issue of joint cartilage thickness. In addition, a new study on ostrich neck flexibility [136] reveals the influence of soft tissue, particularly musculature. In the ostrich, this places greater limits on flexibility than the cervical vertebrae and cartilage alone, suggesting that sauropod necks were less flexible than previously hypothesized and that the animals accordingly had to change their feeding station more often, diminishing the energetic advantage of the long neck.

**Feedback loop.** Reduced vulnerability

Long necks, particularly when their flexibility was limited by cervical ribs [112], [125], [137], would seem to be vulnerable to predator attack and thus be selected against. However, evolutionary increase in body size in adult sauropods beyond the prey spectrum of even the largest theropods would represent an active feedback loop in which the long neck allows larger body sizes, which in turn decreases neck vulnerability [128].

**Trait (new, inferred).** Posterior shift of neck muscles

The importance of the long neck for sauropod gigantism is emphasized by a new trait (inferred), the posterior shift of neck muscles, also observed in extant birds [139]. Already basal sauropodomorphs such as *Plateosaurus* have greatly elongated cervical ribs, extending backwards from the vertebra over two intervertebral joints. Such posteriorly elongated cervical ribs are present in most sauropods, reaching lengths of up to 340 cm [111], with only diplodocoids having short neck ribs (see above). The long ossified cervical ribs of most sauropods suggest a great posterior shift of the hypaxial muscles that attached to them [111], [137].

These muscles either belong to the *m. longus colli* group based on the homology with birds [111], [137], [139], or alternatively, the muscles belong to the *m. scaleni* group based on the homology with crocodiles [112]. Torsion would have been important in the sauropod neck as soon as it was moved laterally, and contralateral activation of these muscles would have efficiently counteracted torsional forces, as it does in modern crocodiles during their “death roll” behavior [112]. Torsional forces would have been particularly pronounced during the lateral movement of a horizontally held neck, consistent with the extreme development of cervical ribs in *Mamenchisaurus* [125]. The torsion hypothesis could be tested by studying long necked-birds that hold their necks horizontally during flight.

**Selective Advantage.** Lightens the neck

Among several beneficial effects of having long ossified cervical ribs [111], [112], the lightening of the neck by moving heavy muscle mass backwards [111], [137] appears particularly relevant in the context of gigantism. This selective advantage acted in concert with the lightening of the neck through diverticula of the respiratory system (see below). Ligthening of the neck probably was one of the contributing factors that facilitated the uniquely elongated neck of sauropod dinosaurs.

### Cascade “Respiration” (Fig. 7)

**Trait (inferred).** Avian-style lung

In recent years, an avian-style respiratory system (ARS, “avian-style lung” in the figures) has become the consensus inference in the respiratory biology of saurischian dinosaurs, including sauropodomorphs [2], [140], [141]. The components of such a system (unidirectional airflow, postcranial pneumaticity, air sacs, and countercurrent gas exchange) do not necessarily depend on each other and could have evolved separately and at different times [142]. Observable evidence, as osteological correlate observed in extant birds, for an ARS is postcranial skeletal pneumaticity (PSP), which now has been traced to the base of Saurischia [37], [142] or even to the base of Archosauria [143], obviating the need for hypothesizing its independent evolution in Sauropodomorpha and Theropoda. Among Sauropoda, specific patterns of PSP, namely the pneumatic hiatus in some neosauropods, is an osteological correlate for thoracic air sacs [144]. In addition, cryptic diverticula (in the sense that they do not leave a trace on the skeleton) probably were widespread in sauropods if not in dinosaurs and ornithodirans in general [144]. Extrem PSP, affecting the distal tail and both limb girdles, was recently described in advanced titanosaurs [144], [145]. Evidence for dorsally attached parts of the lung is also seen in the dorsal vertebral column [140]. Unidirectional airflow, long believed to be unique to birds, has now been documented for living crocodiles as well [142], [146]. Extant phylogenetic bracketing thus would indicate its presence in dinosaurs, including sauropods.

The notion [142] that unidirectional airflow may not be an adaptation to a high BMR because crocodiles have a low BMR is flawed, because the low BMR of crocodilians is likely secondarily derived. The evidence is found in crocodile heart anatomy [147] and in the bone histology of fossil archosaurs that documents a decrease in growth rate from basal crocodile-line archosaurs to crown group crocodiles [148]. In addition, the crocodilian lung “appears overdesigned” [140] for an ectothermic animal. Thus, the combination of high BMR and unidirectional airflow may have been plesiomorphic for archosaurs, with further elaboration of the ARS along the line to birds [140], [143], [147], [149]. This elaboration may well have included a refined counter-current gas exchange system that would have suited the needs of sauropod dinosaurs well [140]. In conclusion, although the sauropod respiratory apparatus may not have been fully homologous to that of birds, its function and advantages must have been very similar.

**Selective advantage.** Lightens the neck

Among the four major selective advantages of an ARS for sauropods, the least obvious but possibly the most important is the effect of the ARS on neck mass. While a light-weight neck would be advantageous at any size, the long, predominantly horizontal neck of large sauropods could only evolve because of PSP, a corollary of an ARS. This statement presumanly applies to long-necked extant birds, long-necked non-avian theropods, and long-necked pterosaurs as well, although this has not been explored in the literature before. The crucial aspect is the development of diverticula of the respiratory tract that invade the medullary region of individual vertebrae. In non-pneumatized bones, this region is filled with bone marrow, but in pneumatized bones it is filled with air.

Pneumatization does not result in a decrease in the mass of the bone tissue *per se*, only in the replacement of bone marrow by air. A pneumatized vertebra thus is lighter than a non-pneumatized one, despite both having the same amount of bone tissue. Statements found even in the most recent literature that “cervical airsacs and extensive cervical diverticula … would also have served to lighten long necks” [111] are not quite to the point in this regard, because it is only the cervical diverticula that lighten the neck, not the cervical airsacs. The diverticula lighten the neck by bringing air into the interior of the neck vertebrae and thus replacing heavy water-rich tissue, i.e., bone marrow, with air. Cervical airsacs exterior to the vertebrae would not have lightened the sauropod neck, they only would have increased its volume without increasing its mass. Current estimates of the specific density of sauropod necks are commonly less than 0.5 [111], based on observed densities of bird necks [111].

In non-avian theropod dinosaurs, the hypothesis that PSP evolved to lighten the skeleton was tested recently [149], and increasing PSP was found to be linked to increasing body mass, corroborating the hypothesis. In sauropods, quantitative tests have not been performed yet, but support is found in the ontogenetic increase in PSP [144]. In light of its importance in the evolution of bird-line archosaurs, PSP deserves further study in extant birds, particularly in regard to its influence on body mass and density.

**Selective advantage.** No dead space problem

The respiratory dead space problem is familiar to human divers and refers to the interdependency of lung volume and tracheal length. If tracheal length is artificially increased (e.g., by a snorkel), tracheal volume may reach a limit where it takes up such a large part of the tidal volume that an insufficient volume of fresh air reaches the lung. The dead space problem affects long-necked animals as well. To avoid the dead space problem, a long neck appears only possible if the non-tracheal ventilated parts of the respiratory system (lungs, air sacs) have a volume that is an order of magnitude larger than that of the trachea. Since the amniote trachea is at least as long as the neck and requires a certain minimum diameter, the long necks of sauropods meant that the non-tracheal ventilated parts of their respiratory system must have been very voluminous [111], [140]. Taylor & Wedel [111] note that sperm whales may have a trachea that is over half of their body length, questioning the importance of the dead space problem for the evolution of a long neck. However, whales as intermittent aquatic breathers may not offer a useful comparative perspective on sauropods, and work on the dead space problem in terrestrial long-necked amniotes is needed.

**Selective advantage.** Continuous oxygen uptake

An unquestionable selective advantage of an ARS is continuous oxygen uptake, as in birds but unlike in mammals, in which oxygen is only extracted during the inhalation part of the breathing cycle. Since the discovery of unidirectional airflow in crocodiles [112], [116], continuous oxygen uptake is present in the extant phylogenetic bracket of sauropods and thus very likely was present in sauropods as well. However, the energetic advantage provided by continuous oxygen uptake compared to inhalation-only uptake still needs to be estimated for sauropods in order to assess the importance of this selective advantage. In extant amniotes, respiration takes up the largest part of the energy budget at rest [150], suggesting that continuous oxygen uptake may confer an important selective advantage, although this needs to be explored further in comparative studies of mammals and birds.

### Cascade “Metabolism” (Fig. 8)

**Trait (inferred).** High BMR

The inferred trait of a high basal metabolic rate (BMR) in sauropods has found additional support by studies published since 2009, but some evidence to the contrary has also emerged.

Comprehensive sampling of ungulate long bone histology, both in terms of taxonomic diversity and of habitat and climate zone [151], revealed the ubiquity of lines of arrested growth in this mammal group, invalidating earlier arguments [152] that the lack of LAGs in mammals *versus* their presence in non-avian dinosaurs indicates different thermophysiologies in the two groups. Improved understanding of the primary bone formation in extant tetrapods led to a refined view of the evidence for high growth rates of sauropod dinosaurs provided by bone histology [153]. Taken at face value, the unusually high density of osteocyte lacunae in sauropodomorphs [154] would suggest a BMR significantly higher than in any other tetrapod group, but this is inconsistent with all other evidence discussed in this section for sauropod BMR having been at the mammalian level or lower. The high osteocyte lacunae density does, however, underscore the uniqueness of this evolutionary lineage. At the microanatomical level, femora of dinosaurs offer additional evidence for a high BMR (“activity metabolism” [155]) in the large nutrient foramina that enter the bone at midshaft: nutrient foramina of extant endotherms (mammals) were significantly larger than those of ectotherms (non-varanid reptiles) because of the lower blood flow to the tissues inside the bone. Non-avian dinosaurs all have large nutrient foramina and the highest estimated blood flow rates to their bone interior among the groups studied [155].

A high BMR requires integumentary insulation structures (hair, feather), at least in small animals. A well preserved small theropod fossil from the Jurassic of Germany [156] now indicates that such integumentary structures were already present in rather basal theropods, narrowing the gap in the fossil record between the integumentary insulating structures occasionally preserved in ornithischian dinosaurs on one hand and feathers on the other[156], making it likely that all dinosaurs, including sauropods, bore such structures, at least as juveniles.

Finally, while research on stable isotopes has long contributed to the endothermy/ectothermy debate, the limitation of this approach remains its proxy nature [157], only indicating temperature of hard tissue formation, not BMR. The new clumped isotope thermometry [157] is a case in point, indicating body temperatures at the endothermic level for sauropods, but these could have resulted from thermal inertia (“gigantothermy, mass homeothermy”) as well. Thermal inertia, however, would not have supported the active lifestyle of sauropods and other dinosaurs that is indicated by their upright stance (see below), because a new study on large crocodiles indicates that their power output is an order of magnitude less than that of similar-sized mammals [158].

Body temperatures can also be calculated from maximum growth rates [159], [160]. These studies suggest that in dinosaurs, unlike in crocodiles, body temperature did not increase with body mass, inconsistent with thermal inertia or mass homeothermy. In fact, these studies [159], [160] infer a body temperature decrease with increasing body mass for sauropods, suggesting that they had an efficient cooling system to prevent overheating [160]. Absolute body temperatures in sauropods calculated from maximum growth rates are lower than expected for a similar-sized mammal, possibly indicating a lower BMR [160], but still relatively high.

While there is thus strong evidence that sauropod dinosaurs had a BMR at least in the lower range of large mammals but possibly higher, a new study on growth rates [150], discussed below, questions this conclusion.

**Feedback loop.** Low mass-specific metabolic rate

The negative allometry of BMR with body mass (see [100], [161], [162] for a discussion of this scaling relationship) means that larger animals need to take up less energy per unit body mass to enjoy the benefits of a high BMR. This effect represents a feedback loop from the trait “Very large body mass” to the trait “High BMR”.

**Feedback loop.** Heat loss through long neck

A classical argument against a high BMR in sauropods has been the overheating problem faced by very large endothermic animals because of their poor surface to volume ratio. This would have limited the surface area through which the excess heat generated by the animal could have been dumped via radiative and convective heat loss [163], [164]]. Mechanisms such as the active control of blood flow from the body core to the body surface, as observed in crocodiles [165], auxiliary integumentary features such as the African elephant's large ears, and nocturnal loss of heat stored during the day [164] are difficult to reconstruct for sauropods. However, the unique sauropod body plan with the long neck and long tail had a more favorable surface to volume ratio than a sauropod-sized elephant or rhino. In particular, positive allometric scaling of neck surface area with basal metabolic rate is consistent with a heat loss function of the neck [126].

A long neck also plays a role in heat loss through an avian-style respiratory system, as discussed below [166]. The long neck was thus part of a positive feedback loop, in which it supported the high BMR of sauropods through its role in thermoregulation (Fig. 8).

**Selective advantage.** Heat loss through ARS

In the ECM, heat loss is also hypothesized to have been a selective advantage of an ARS beyond its other roles in facilitating the long neck of sauropods. Thus, an ARS and a long neck would have acted in concert in the dumping excess heat (Fig. 8).

The respiratory system of extant birds is well known to function in body temperature control, raising the question whether this function was served by the ARS hypothesized for sauropods [140], [141]. A novel modeling approach, computational fluid dynamics (CFD), can be used to assess the function of the ARS in heat loss [166]. A two-dimensional CFD model of heat exchange in the trachea and air sacs of domestic chicken was used to validate the method [166]. A three-dimensional CFD simulation of the respiratory tract of a sauropod would serve to test the hypothesis.

**Selective advantage.** Fast conversion of energy from environment

No new studies relevant to sauropod gigantism have been published that address the selective advantage of a high BMR, i.e., the fast conversion of energy from the environment, which in turn appears necessary for high growth rates. However, this fast conversion of energy from the environment is implicit in the most widely accepted hypothesis of the origin of endothermy, the aerobic scope hypothesis [167].

**Trait.** High growth rate

Unlike a high BMR, which must be inferred, growth rates can be calculated in non-avian dinosaurs based on growth marks in their long bones. While growth rates have been well constrained in theropods and ornithischian dinosaurs [168], [169], [170], sauropod growth rates have been difficult to estimate [171], and seemingly inflated growth rates of >5000 kg per year continue to be perpetuated even in the most recent literature [34], [150], [171]. A global view of dinosaur growth rates, using local tissue apposition rates as proxy, suggests that growth rates an order of magnitude higher than in living reptiles evolved in early dinosaurs and remained high throughout the group [148]. The important question regarding the trait “High growth rate” is comparative, i.e., how do sauropod growth rates compare to those of living reptiles, mammals, and birds.

A first set of comparative data for growth rates in non-titanosaurian sauropods based on long bone histology is now available [89], and a single but well constrained data point was derived from growth marks in ribs [172]. These studies indicate that non-titanosaurian sauropod growth rates were in the realm of scaled-up modern ratite birds and mammalian megaherbivores, but were lower than the average mammal [89]. Titanosaur growth rates still have defied quantification, but qualitative evidence from long bone histology (i.e., modified laminar bone) suggests a phylogenetic reduction in growth rates in many smaller titanosaurs [173], [174], albeit not accompanied by a reduction in BMR [173], [174].

In general, growth rate data for sauropods remain more poorly constrained than for any other dinosaur group that has been sampled histologically to any extent because of the rarity and poor development of growth marks in sauropod long bones [173], [174]. Growth rate estimates based on the growth mark record thus probably represent minimum growth rates [171].

The link between maximum growth rate (MGR) and BMR in vertebrates was first explored by Case [90], who calculated regression lines for major extant vertebrate groups and noted that terrestrial endotherms (mammals and birds) have an order of magnitude higher MGRs than ectothermic amniotes. Surprisingly, this link between MGR and BMR has received little attention since, not even from the perspective of the metabolic theory of ecology. In a new study, Clarke [150] compared dinosaurian MGR with those of extant mammals and reptiles, using the dataset of Case [141]. The regression for dinosaur growth rates, including those of sauropods, was intermediate between those for mammals and reptiles. Clarke [150] then entered the comparative data on growth rates into a model of the energy budget of various dinosaurs and concluded that most of the observed growth rates could have been achieved with a reptilian energy budget and BMR, concluding that this evidence made a high BMR in non-avian dinosaurs unlikely.

There are several points in the approach of Clarke [150] that require modification and further work, if it is to serve as a test of the trait “High growth rate”. For one, the Case dataset [90] is not up to date and could be replaced by a current one, which is availabe in the literature. Also, there are no large fast-growing non-avian reptiles, placing the data points for all sauropodomorphs outside the point cloud for non-avian reptiles, and a separate comparison of sauropodomorphs and mammals should be done. Finally, as already noted, current estimates of sauropod growth rates probably underestimate true rates considerably. Nevertheless, a certain contradiction remains between the evidence for high growth rates from bone histology [148], [171] and lower growth rates from modeling of growth [160] and energy budget [150]. The influence of parental energy transfer on MGR remains poorly understood as well and should be studied in extant animals. Any kind of parental care, even simple guarding behavior, represents an energy transfer from parent to offspring, increasing offspring growth rate. With sauropods presumably lacking any form of parental care (see above), their offspring was fully autonomous, possibly limiting its growth rate as well as our ability to predict BMR from MGR.

In conclusion, the trait “High growth rate” in the evolutionary cascade has not been falsified because all studies agree that sauropod MGR experienced a manifold evolutionary increase compared to their closest living and non-dinosaurian extinct relatives.

**Selective advantage**. High likelihood to survive to adult stage

Independent of the necessity of a high BMR to achieve fast growth, the selective advantage of a high growth rate appears clear. Especially in animals that, like no other amniote, had an extreme size difference between embryo and adult [12], [14], [52], fast growth to survive to adulthood would have been of great selective advantage, considering the formidable predation pressure faced by juvenile sauropods. Such fast growth has recently been detected in embryos of the basal sauropodomorph *Lufengosaurus* [175] and has been suggested to indicate extremely fast growth in the hatchlings as well [175]. This selective advantage would be easy to test in extant animals, and tests may well be already contained in the zoological literature.

**Trait.** Upright stance

All large extant terrestrial tetrapods with a high BMR have an upright stance [132]. This limb posture is required for the energy-efficient mode of parasagittal locomotion in which the limbs function according to the principle of the inverted pendulum [104]. An upright stance is a derived characters at the level of Dinosauria, and the upright stance was a prerequisite for sauropod gigantism not only because it preceded the graviportal stance of sauropods [176], [177], [178], [179] but because of its link with a high BMR. Parasagittal locomotion is necessary for large animals with a high BMR to acquire enough energy from their environment to support this high BMR which, in turn, allows continuous locomotion. Thus, the causation and its direction in these two traits is not sufficiently understood (Fig. 8).

**Selective advantage.** Energy-efficient locomotion

Energy-efficient locomotion as a selective advantage resulting from the upright stance was discussed above, but the question could be asked whether sauropods were more efficient locomotors than extant graviportal mammals and other graviportal dinosaurs. Locomotion in sauropods can be understood from two independent and complementary lines of evidence: their skeletons and their rich track record. More efficient locomotion has not figured in previous hypotheses about sauropod gigantism, but further considerations are in order. Specifically, can we formulate hypotheses that posit that any aspect of the locomotory apparatus and locomotion facilitated the unique gigantism of sauropod dinosaurs? In particular, are there any scaling factors in locomotion that would favor larger body size over smaller body size? Negative allometry of the cost of transportation might be one such factor but it could not be detected in the study by Preuschoft et al. [128].

Future research concerning the hypothesis of “energy-efficient locomotion” could be based on quantitative biomechanical models, but it will require an improved understanding of sauropod gaits. These have not been reliably reconstructed, neither from models nor from theoretical considerations [128]. The latter study [128] excluded all gaits with a suspended phase and all asymmetrical gaits. Current quantitative research on sauropod footprints using different approaches may improve this situation [78], [180], [181]. Such research also needs to include studies on extant animals with an upright stance with the aim of reconstructing gaits from trackways (e.g., [182]). Good starting points would be horses and elephants.

## Discussion

### Revised ECM for Sauropod Gigantism

**Figure 9:**
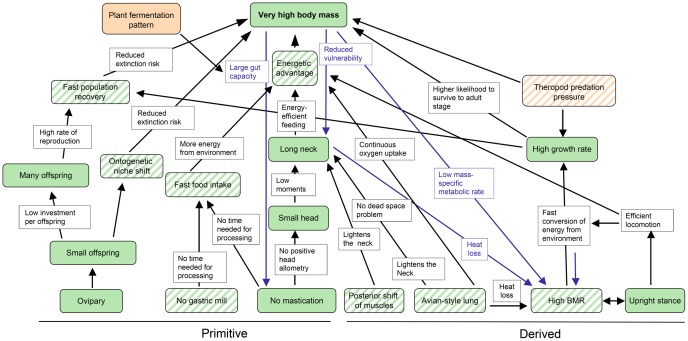
Revised ECM for sauropod gigantism. Conventions used are the same as in Fig. 1, except that a distinction is made between observed traits and premises (solid color) and inferred traits or premises (oblique stripes). Compared to the original ECM (Fig. 1), complexity has increased considerably as has integration, with each cascade being connected with at least one other cascade. Note the central position of the cascade “Head and neck” and the many arrows pointing at the traits “Long neck” and “Energetic advantage”. See text for further explanations.

The remarkable amount of evidence that has accumulated over the last few years, and that is the focus of this collection, considerably refines the evolutionary cascade model of sauropod gigantism proposed by Sander et al. in 2010 [2] by testing many of its components. The ECM has become more complex with the splitting of cascades, the addition of traits, and the addition of links between cascades, i.e., selective advantages and feedback loops (Fig. 9). Many of the inferred traits and hypothesized selective advantages have found support. A minority were falsified or at least called into question, without affecting the overall picture, however.

Compared to the 2010 ECM, the cascade “Reproduction” has been refined by splitting the basal trait “Many small offspring” into three different traits and by adding a subcascade that takes into account the ecological effects of the body size difference between hatchlings and adults (Fig. 4). The cascade now appears to be better supported than ever since its origin in the work of Janis & Carrano [49].

The original cascade “Feeding, Head, Neck” has also been split into two cascades, “Feeding” (Fig. 5) and “Head and neck”(Fig. 6) that are linked to each other in the trait “No mastication”. New evidence supports all traits in the cascade, including the lack of a gastric mill. However, while the hypothesis that mastication limits food intake rate has received further support, the same limitation may not apply to a gastric mill, contrary to the original ECM. One aspect of the feedback loop “Large gut capacity”, i.e., the positive scaling of food retention time with body mass (“Jarman-Bell Principle”) may not hold up [100]. This research offers an example of how work on sauropod dinosaurs can question long held views on the biology of extant animals.

The cascade “Head and neck” (Fig. 6) probably has received the most attention because researchers have come to fully appreciate the central importance of the neck in sauropod biology and evolution. New modeling approaches and a refined understanding of neck anatomy (e.g., the function of cervical ribs) have strengthened and refined this cascade, leading to the addition of the inferred trait “Posterior shift of muscle bulges” and the selective advantage of “Lightening the neck” (Fig. 6). Similarly, the cascade “Avian-style lung” has been strengthened by further evidence but without experiencing modifications (Fig. 7).

The cascade “High BMR” was amended by adding “Upright stance” as an observed trait and “Efficient locomotion” as the selective advantage (Fig. 8). Much new evidence in support of this cascade has accumulated and hypothetical selective advantages have been tested, but there is also contradictory evidence. Specifically, the trait “High growth rate” has been called into question by growth rates calculated from bone histology, while at the same time other evidence from bone histology strengthens the case for fast growth in sauropods at the mammalian level (Fig. 8). In addition, the trait “High growth rate” is important for the trait “Fast population recovery”, which had been recognized before [2] but not visualized in the original sauropod gigantism ECM.

### Status of the ECM and future improvements

The ECM for sauropod gigantism is of heuristic value for explaining the unique body size of sauropod dinosaurs and the limits to body size in terrestrial amniotes in general. However, the ECM currently does not provide information about the relative contribution of the component cascades and their basal traits to gigantism (see also[2]) and if any of the traits were a necessity for sauropod gigantism. Thus, we do not know whether ovipary was more important than a high BMR or than the lack of mastication (see also the ternary diagram in Sander et al. [2]). One way to improve this situation would be to take the energetic approach to sauropod gigantism [2] to its logical conclusion by modeling the energy budget of a living sauropod dinosaur, following the approach of Clarke [150]. This is suggested by the observation that four of the cascades indicate an energetic advantage as an explanation for gigantism. The other way of testing the ECM will be to bring a phylogenetic approach to it, including character optimization, character correlation analyses, and phylogenetic comparative methods. By comparing the presence or absence of these traits in other terrestrial amniotes with their maximum body size, we can estimate the relative importance of traits, but without quantification [2]. The revised ECM allows a refined understanding of body size limits in other terrestrial amniotes beyond the discussion in Sander et al. [2].

### Limits to terrestrial amniote body size

This discussion of the limits to body size is restricted to terrestrial amniotes here because so many parameters are different in the marine realm (trophic structures, cost of transport, heat conduction of medium, etc.) that meaningful comparisons are not obvious. Terrestrial amniotes show the following maximum body size distribution: the largest non-avian reptiles (three clades) and birds are smaller than the largest mammals; these are smaller than the largest theropod and ornithischian dinosaurs, which in turn are smaller than the largest sauropod dinosaurs. Except for non-avian reptiles, the largest (or all) species in these clades are herbivores and are an order of magnitude larger than the largest carnivorous members of their respective clades. In addition, studies of how body size is distributed across the size range of the clade [9], [12] shows that sauropods differ from the other clades in that most sauropods are large. Ornithischians show a less pronounced left skew in body size distribution while mammals and birds show a strong right skew [9]. However, these studies [9], [12] may suffer from the difficulty of comparability of the clades involved.

A number of factors can be identified limiting body size based on recent research and the ECM (Table 1), but a few invite further comments. The limit to body size in sauropods may well have been set by the design of the tetrapod skeleton in combination with the scaling of muscle power to body mass.

**Table 1:** Factors limiting body size in terrestrial herbivorous amniotes.

Sauropoda:
- Scaling of locomotory muscle power with body mass [189]
Ornithischia:
- Mastication, limiting food intake rate and neck length [100]
- Possible lack of internal respiratory cooling capabilities [166]
Mammalia:
- Mastication, limiting food intake rate and neck length [100]
- Lack of internal respiratory cooling capabilities [166]
- Reproductive output [14]
Reptilia (non-dinosaurian):
- Low BMR and low growth rate [2], [14]
Aves:
- Parental care combined with ovipary [71]
- Possibly hindleg design [186]

Taxa are arranged in order of decreasing maximum size and increasing right skew of body size distribution. References are to the most recent papers only.

Mastication-induced positive head allometry, as predicted by scaling principles, is documented for ornithischian dinosaurs by a recent study of ontogenetic changes in the skull of a hadrosaur species [114]. The strongly positive snout allometry in this dinosaur is consistent with hadrosaurs being highly efficient chewers as shown by the complexity of their dental tissues [183].

The question of why no multi-tonne ground birds evolved in the early Tertiary after the demise of the non-avian dinosaurs remains prominent [184], considering that birds seem to show all of the traits in the revised sauropod gigantism ECM in which a gastric mill, obligatory in herbivorous birds, is not necessarily seen as limiting food intake rate (see above). Explanations are sought in features of the locomotor system and reproduction of birds that have evolved beyond the state in non-avian dinosaurs [184]. The most obvious difference is sauropod graviportal quadrupedalilty vs. bird bipedality. In addition, bird hind leg posture and musculature differ from non-avian dinosaurs in that the femur is held subhorizontally, and the retraction of the leg is mainly achieved in the knee joint [178], [185]. Reproduction of avian dinosaurs includes brooding and parental care, features that evolved in the most derived non-avian dinosaurs [186]. These led to a different scaling of egg size with body mass in birds [71] than in less derived dinosaurs, meaning that the upper limit of egg size apparently was reached in birds at a body mass of less than 1000 kg [71]. Body size of other extant oviparous amniotes such as turtles, lepidosaurs, and crocodiles apparently was not limited by their mode of reproduction but by a low metabolic rate [2], [14].

As shown by this example of birds and extant non-avian reptiles, but also by the many other taxa and traits in Table 1, the evolution of maximal body size is often constrained by historical contingency. Traits that were highly adaptive for a lineage at small body size constrained maximum body size of the lineage later in its evolution. Only by taking the comparative approach to as many extinct and extant lineages as possible, these constraints can be understood. The study of dinosaur gigantism thus becomes a research program of general relevance in vertebrate evolutionary biology. Note that the sauropod gigantism ECM thus makes predictions about the future evolution of lineages, such as that mammals are unlikely to ever evolve the body size of sauropod dinosaurs.

**Figure 10:**
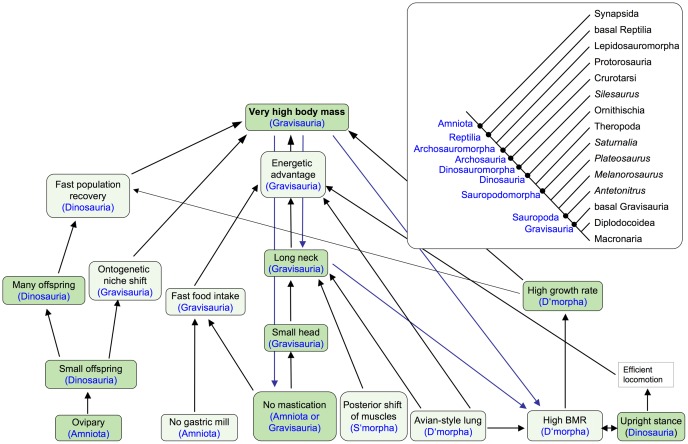
Phylogenetic distribution of traits in the sauropod gigantism ECM. For each trait in the model, the likely inclusive taxon in which the trait evolved is indicated. Note that Gravisauria is the taxon in which most of the classical sauropod traits appear. Darker green traits are observed, lighter green traits are inferred. Black arrows indicate evolutionary causation and blue arrows indicate feedback loops.

Beyond the notion that some in the ECM are plesiomorphic and some are derived, the question can now be addressed of when the basal traits of each cascade arose in the phylogeny and how this conincides with body size increase. As noted earlier, it will be difficult to bring these two datasets into perfect congruency because of the difficultiy of plotting the largest sauropodomorph remains from any time bin onto the phylogeny. While the traits “Ovipary”, No gastric mill”, and “No mastication” are plesiomorphic for amniotes (Fig. 10), the avian-style lung probably evolved at the base of Dinosauria [37]. The trait “High BMR” also evolved at the base of Dinosauria [148] The trait “Posterior shift of muscles” in the neck was present in basal sauropodomorphs such as *Plateosaurus*, as evidenced by greatly elongated cervical ribs and their histology [112]. Greatly enlongated neck ribs together with neck elongation by elongation of individual vertebrae is alsready seen in basal archosauromorphs such as the Late Permian *Protorosaurus* [187], but the evolution of neck ribs in archosauromorphs has not been documented in sufficient detail to exclude convergent evolutionThe other traits in the ECM (Fig. 10) can also be mapped on the sauropodomorph cladogram, although this aspect of the ECM requires additional research.

Optimizing traits from the ECM onto a phylogeny that includes all the terminal taxa which exhibit the trait will be a fruitful avenue to explore. The ultimate test of the importance of the presumed factors in the evolution of amniote body size would be to test their contribution to body size across amniotes, using phylogenetic comparative methods.

## Conclusions

This review of the biology of the sauropod dinosaurs and the evolution of their gigantism, condensed into the sauropod gigantism ECM, serves to compile and synthesize the rapidly expanding literature on the subject, including this collection in PLoS ONE. It also serves as an update to an earlier review [2] in which the evidence available in late 2009 was synthesized into a unified biological scenario of sauropod gigantism, using the approach of an evolutionary cascade model. Testing the premise that it is mainly intrinsic factors rooted in the biology of the clade Sauropodomorpha that explains the historical pattern of its evolution to gigantic body size, was no the aim of this review. However, the evidence reviewed here shows at least that there is no need to invoke extrinsic, abiological factors to explain sauropod gigantism. Testing the influence of environmental change over geological time scales on the historic pattern of evolution is a valid research program, but it is not the one we pursue.

The rich new evidence accumulated in these last four years was then used to test the ECM by asking how this evidence impacted the component cascades and the entire ECM. Most of the inferred traits, selective advantages, and feedback loops in the ECM found support, sometimes strongly so, while in a few others (e.g. “High growth rate”) support weakened or relationships had to be rejected (the physiological underpinning of the feedback loop “Large gut capacity”). The ECM was also refined by splitting up traits and adding new ones. The general conclusion of Sander & Clauss [1] and Sander et al. [2] that sauropod gigantism was able to evolve because of the complex interplay of a historically contingent combination of plesiomorphic (primitive) and derived traits and characters, has emerged stronger than before. While the principle of parsimony calls for preference of simple solutions over complex ones, it is simplistic to assume that a single factor will explain sauropod gigantism. Finally, the sauropod gigantism ECM is hoped to evolve into a comprehensive framework informing us about evolutionary body size limits in herbivorous tetrapods in particular and other terrestrial tetrapods in general.
